# Enhanced Thermal Properties of Carbon-Based Composite Phase Change Materials Towards Energy Efficiency

**DOI:** 10.3390/ma19183960

**Published:** 2026-09-18

**Authors:** Haipeng Li, Xiang Liu

**Affiliations:** 1 State Key Laboratory of Intelligent Construction and Healthy Operation and Maintenance of Deep Underground Engineering, China University of Mining & Technology, Xuzhou 221116, China; 2 School of Chemical Engineering and Technology, China University of Mining & Technology, Xuzhou 221116, China; cumtgccs@163.com

**Keywords:** energy-saving, shape-stabilized phase change material, temperature regulation, thermal energy storage

## Abstract

Carbon-based shape-stabilized phase change materials (SSPCMs) are promising for thermal energy storage, but their performance is often limited by low PCM loading. Enhancing the compatibility between the PCM and supporting matrix is an effective approach to address this issue. Herein, biomass waste was converted into activated biochar supports through chemical treatment with different agents, a process that simultaneously regulated the construction of micro- and mesopores and enriched surface functional groups. This led to optimal compatibility and interaction with lauric acid (LA) and myristic acid (MA). The prepared SSPCM demonstrates excellent leak-free performance with a high PCM loading of 81%. The composite also exhibits a high thermal energy storage capacity, with melting and crystallization enthalpies of 134.0 J g^−1^ and 106.4 J g^−1^, respectively. Furthermore, an SSPCM-based coating displays effective temperature regulation in a simulated indoor environment. This work provides a new insight into the design of cost-effective, high-performance thermal storage materials.

## 1. Introduction

With the sustained growth in global energy demand, improving energy efficiency and reducing energy consumption have emerged as a pressing challenge. Buildings account for approximately 30% of total global energy consumption, and 50~60% of overall building energy consumption is consumed by heating and cooling systems that maintain indoor thermal comfort [1]. Conventional indoor thermal regulation mainly depends on high energy-consuming equipment, which not only intensifies pressure on energy supplies but also increases greenhouse gas emissions. Therefore, developing new energy-saving materials to reduce building energy consumption is crucial for energy saving and emission reduction [2]. Phase change materials (PCMs) have attracted growing attention owing to their capability to regulate thermal energy via absorbing and releasing heat during phase transitions [3,4,5]. PCMs have presented favorable engineering feasibility and cost-effectiveness in applications such as solar thermal energy utilization, thermal management of electronic devices, and cold-chain logistics, and they are particularly promising for building energy conservation [6]. Integrating PCMs into building envelopes, such as walls and roofs, can effectively buffer indoor temperature fluctuations and reduce reliance on mechanical heating and cooling equipment, thereby promoting energy savings and reducing CO_2_ emissions.

PCMs can be broadly classified into inorganic and organic types, including paraffin wax (PW), polyethylene glycol (PEG), fatty acids, and so on. Among organic PCMs, fatty acids are regarded as a particularly attractive option for building energy conservation and low-temperature thermal storage due to their abundant feedstock availability, environmental friendliness, high latent heat of fusion (typically 153~182 J g^−1^), and a phase-transition temperature range of 20~70 °C [7,8]. However, single-component fatty acids exhibit problems such as fixed melting point, subcooling and phase segregation during repeated thermal cycling, which limit adaptability and reliability under diverse operating conditions. Accordingly, binary eutectic design has emerged as an effective strategy [9,10]. By regulating melting point and crystallization behavior through intermolecular interactions, it can significantly reduce overcooling and improve cycle stability and thermal reliability, while maintaining high latent heat as much as possible. Among the various eutectic systems, the binary system of lauric acid (LA) and myristic acid (MA) is particularly typical [11]. Its eutectic transition temperature is usually about 23~40 °C, aligning well with the human thermal comfort range and building habitable temperatures. The pure LA-MA eutectic (66.0 wt% LA) exhibits a relatively high latent heat of fusion of 166.8 J g^−1^ with a melting point of 34.2 °C [12], and an even higher value of 177 J g^−1^ has been reported for the same eutectic system [13]. These characteristics make it well suited for applications such as PCM-enhanced wall insulation, low-temperature floor heating, and passive thermal management in buildings.

The inherent defects of PCMs, such as phase separation and leakage, limit their practical application and reduce energy utilization [14], thus the concept “shape-stabilized phase change materials (SSPCMs)” has been proposed [15,16,17], in which the skeleton material encapsulates PCMs. Relying on capillary force and interface interactions, the skeleton can confine the molten PCMs and limit their leakage. It is worth noting that skeleton materials derived from natural mineral resources or artificial skeleton materials made from natural resources can destroy the local ecological environment. Microencapsulation technology, which has developed rapidly in recent years, has also prevented the leakage to a certain extent by encapsulating PCMs within a polymeric shell [18]. Nevertheless, the reliance of such techniques on petrochemical-derived raw materials not only increases fabrication cost but also imposes additional environmental burdens.

At present, the use of carbon-based materials as a skeleton for fabricating SSPCMs has been demonstrated to be effective in restraining leakage. Compared with ordinary carbon materials, biochar shows obvious advantages, such as abundant raw materials derived from biomass waste, and has large specific surface area, well-developed porosity, and rich functional groups, making it highly suitable as a support framework for shape-stabilized PCMs [19,20,21]. Additionally, carbon-based SSPCMs derived from biomass not only retain the high energy-storage density and temperature-regulation capability of PCMs, but also benefit from the porous biochar skeleton, which can enhance thermal stability. Multiple studies have demonstrated that biochar effectively suppresses leakage of fatty acids or paraffin and maintains geometric stability in sheet-, block-, or coating-type configurations [22]. Moreover, Zhang et al. [23] used a palmitic acid–lauric acid eutectic mixture as a PCM and carbonized waste rice as a skeleton material to prepare an SSPCM, and the loading capacity of the PCM is 78.2%. Atinafu et al. [24] obtained three types of biochar from pyrolysis of straw, seed and sewage sludge, and biochar prepared from seed with a large specific surface area (77.6 m^2^ g^−1^) has the highest loading capacity for 1-dodecanol and n-dodecane, demonstrating high energy storage potential.

Furthermore, improvement in heat-storage capacity remains to be addressed in current research on SSPCMs. Activated biochar is obtained through physical and chemical methods to improve the loading amount of PCMs, thereby increasing the phase change enthalpy of SSPCMs. Chin et al. [25] prepared palm kernel shell activated carbon through steam activation and further fabricated a composite SSPCM with a paraffin loading capacity of 31%. The results showed that the melting and solidification temperatures are 29.2 °C and 31.6 °C, respectively, with corresponding latent heats of 57.3 kJ kg^−1^ and 57.2 kJ kg^−1^. Hekimoğlu et al. [26] used walnut shells to prepare biochar via pyrolysis and ZnCl_2_-activated biochar, and the loading capacities for methyl palmitate are 43% and 55%, respectively. Yin et al. [27] also reported that ZnCl_2_-activated biochar was then oleophobically modified, and the modified activated carbon had a paraffin wax loading capacity of 84.26%. In short, preliminary research shows that activated biochar has higher porosity, larger specific surface areas, and better adsorption properties, making it more suitable as a skeleton material for PCMs.

The development of eco-friendly and low-cost skeleton materials has increasingly become a key research focus. Currently, research on biochar as a skeleton material mainly focuses on direct carbonization of biomass in an inert atmosphere, while the activated biochar-based SSPCMs with better performance for PCM loading are relatively scarce. In addition, the latent heat storage performance of activated biochar-supported fatty acids has not yet been sufficiently explored, which constrains the broader adoption of SSPCMs in engineering practice for enhancing thermal regulation performance in buildings. This study developed a novel carbon-based shape-stable phase change composite material. Waste coconut shell was used as raw material to produce activated biochar as a new skeleton material via carbonization followed by chemical treatment, then LA and MA eutectic mixture as the PCM was loaded into the porous carbon matrix using a vacuum impregnation method. The three-dimensional porous carbon achieves excellent loading and stable immobilization of LA-MA through pore filling, hydrogen-bond anchoring, and interfacial affinity. In addition, a comprehensive evaluation of the obtained SSPCM was comprehensively evaluated in terms of phase transition enthalpy, thermal stability and temperature regulation performance. This study deepens our understanding of the correlation between the microstructural characteristics and the thermal properties of carbon-based SSPCMs and potentially contributes to the optimization of energy-efficient SSPCMs in buildings.

## 2. Materials and Methods

### 2.1. Materials

Myristic acid (MA, 98.0%) and lauric acid (LA, 99.0%) from Sinopharm Chemical Reagent Co., Ltd. (Shanghai, China), were used as PCMs, and their properties are summarized in Table 1. Coconut shells were obtained from Hainan Province, China. Proximate analysis was performed according to the Chinese national standard GB/T 28731-2012 [28] to characterize coconut shells. The corresponding data are presented in Table 2. In addition, KOH, MgCl_2_, CaCO_3_ and ZnCl_2_, all of analytical grade, were supplied by Sinopharm Chemical Reagent Co., Ltd. (Shanghai, China). Putty powder was purchased from Inner Mongolia Yaoya Decoration Co., Ltd. (Hohhot, China).

### 2.2. Eutectic Point of Binary Phase Change Materials

Although the LA-MA eutectic composition has been widely reported, the measured values can vary considerably with raw-material purity and thermal-analysis conditions. Since the subsequent experiments were all based on the fatty-acid raw materials purchased in this study, the eutectic ratio was re-determined by our own analysis.

**Figure 1:**
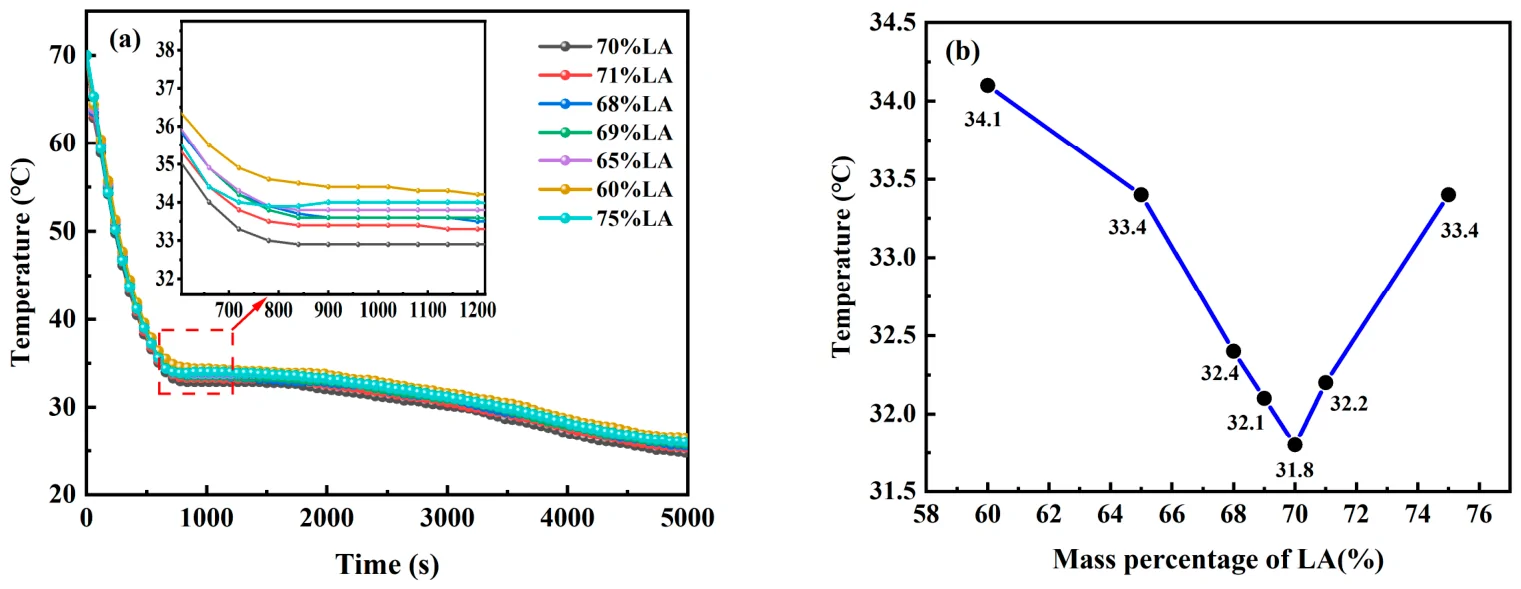
(**a**) Step-by-step cooling curves of LA-MA with different mass fraction ratios; (**b**) the partial phase diagram of LA-MA.

The eutectic point was determined using the step-cooling curve method [29], which is a common analytical method. Briefly, test tubes containing the PCM were uniformly heated in a thermostatic water bath until complete melting. Once the PCM temperature stabilized at 70 °C, the tubes were transferred to a constant-temperature chamber set at 25 °C to allow cooling and crystallization. Temperature data were recorded at 1 s intervals using the sensor until the temperature reached a steady state. The temperature versus time diagram of the PCM during the cooling process is the step-cooling curve. The experimental measurements indicate that the eutectic composition of the binary PCM is within the range of 60~75 wt% LA. Step-by-step cooling curves of LA-MA and the results are summarized in Figure 1. As shown in Figure 1a, when the LA mass fraction was 60%, 65%, 68%, 69%, 70%, 71%, and 75%, the corresponding phase change temperatures were 34.1 °C, 33.4 °C, 32.4 °C, 32.1 °C, 31.8 °C, 32.2 °C, and 33.4 °C, respectively. Figure 1b presents the partial phase diagram of LA-MA system. As shown, when the mass fraction of LA increases from 60% to 75%, the phase change temperature of the mixture decreases initially and then increases. The minimum phase change temperature occurs at an LA mass fraction of 70%, indicating that this composition corresponds to the eutectic composition.

The LA-MA eutectic obtained in this work shows a slight deviation from the reference, which reports LA contents of 58~66 wt% and melting points between 33.3 °C and 35.2 °C [30]. This deviation can be mainly attributed to differences in feedstock purity as well as variations in the measurement conditions.

### 2.3. Fabrication of SSPCMs

**Figure 2:**
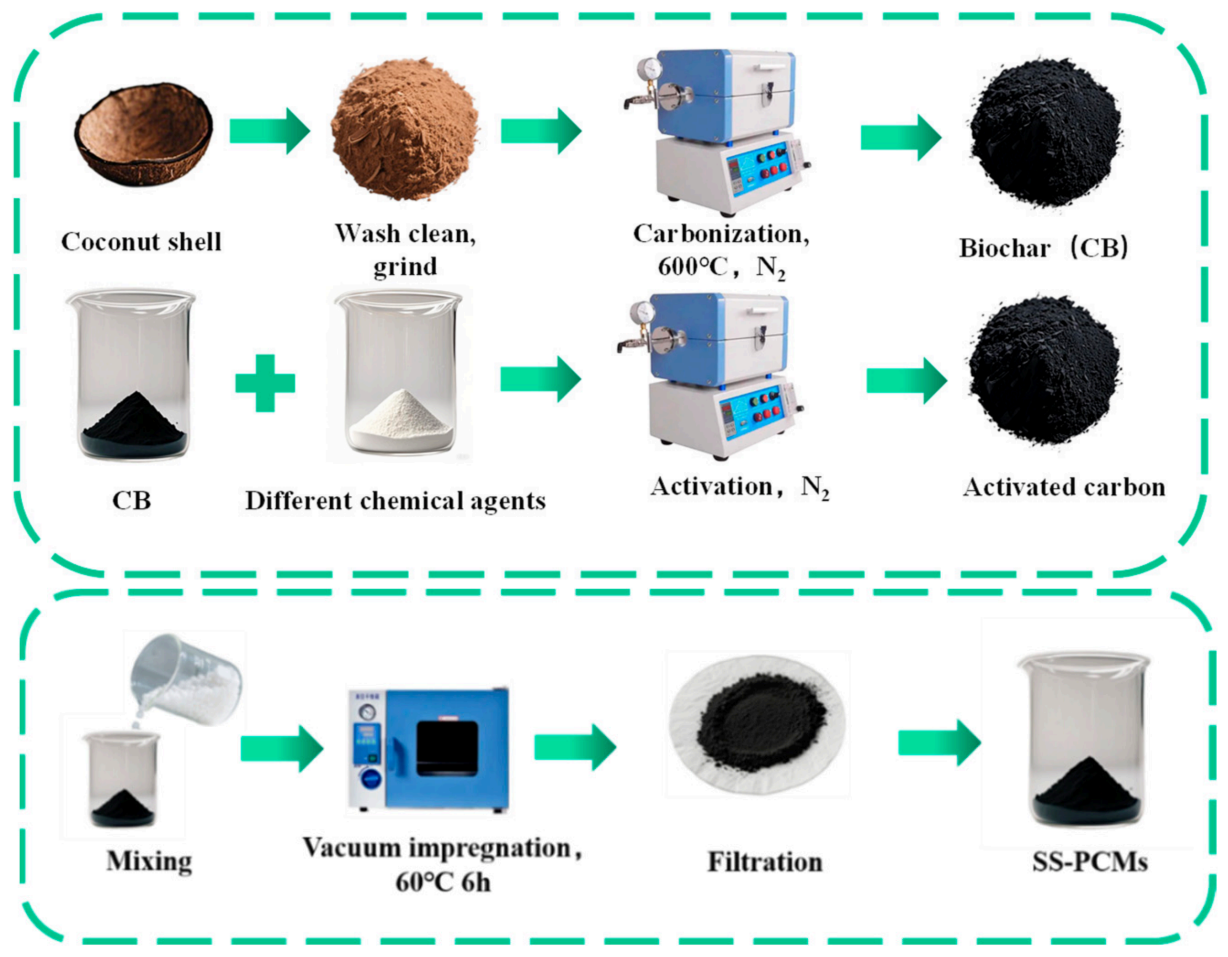
Schematic illustration of preparing activated biochar/LA-MA.

The synthesis procedure for activated biochar/LA-MA SSPCMs is illustrated in Figure 2. The biomass-waste coconut shells were thoroughly washed and oven-dried in an oven at 60 °C for 3 h. After crushing and milling, the powder was sieved to 200 mesh and placed in a porcelain boat, which was then transferred to a tubular activation furnace (OTF-1200X, Hefei Jingke Materials Technology Co., Ltd., Hefei, China). Carbonization was conducted under an N_2_ atmosphere at 600 °C for 2 h, followed by natural cooling to room temperature to obtain the carbon (denoted as CB).

CB was further mixed with one activating agent (KOH, ZnCl_2_, MgCl_2_ and CaCO_3_) at CB-to-agent mass ratios of 1:1~1:4. Under an N_2_ atmosphere, the mixture was heated to 600~750 °C and activated for 2 h. During the heat treatment, pores were generated by the agent either reacting with the carbon source or serving as a sacrificial template. The ratio range was based on the commonly reported activation ratio (typically 1:1~1:5), which generally produces carbons with a high specific surface area. After cooling, the product was first acid-washed and then repeatedly treated with deionized water until neutral. The solid was dried at 60 °C for 24 h in vacuum to obtain the carbon-based support. At a CB-to-agent mass ratio of 1:3, the samples were denoted as KCB, MCB, CCB and ZCB, for KOH, MgCl_2_, CaCO_3_ and ZnCl_2_ respectively.

Lauric acid and myristic acid binary eutectic mixture (LA-MA) with the LA mass fraction of 70% was prepared in advance. For each sample, 10 g of the mixture was placed in a flask. The samples were heated at 60 °C and stirred for 1.5 h to ensure complete melting, then carbon-based support was added. A vacuum impregnation method was employed to fabricate the shape-stabilized PCM. The flask was transferred to a vacuum oven maintained at 60 °C for 6 h. After impregnation, the samples were removed and placed on filter paper and subsequently kept in an oven at 60 °C for 1 h to eliminate residual PCM on the surface. The filter paper was replaced periodically until no further PCM leakage was observed, thereby yielding the SSPCMs derived from activated biochar.

### 2.4. Characterization Methods

The surface morphology of the samples was evaluated by MAIA3 FSEM (TESCAN, Brno, Czech Republic). FTIR measurements were performed using a Thermo Scientific Nicolet 380 infrared spectrometer (Thermo Fisher Scientific Inc., Waltham, MA, USA). X-ray photoelectron spectroscopy (XPS) characterization was performed on a Thermo Fisher ESCALAB 250Xi (Thermo Fisher Scientific Inc., Waltham, MA, USA) equipped with Al Kα radiation and the C1s binding energy of 284.8 eV was used as the internal standard to correct the shift in binding energy. N_2_ adsorption–desorption was used to evaluate the pore characteristics using Autosorb-IQ2-MP-XR (Anton Paar GmbH, Boynton Beach, FL, USA). The specific surface area was derived from the BET method, and the pore size distribution and total pore volume were analyzed by the DFT model. Differential scanning calorimetry (DSC) was performed using NETZSCH DSC 300 (NETZSCH-Gerätebau GmbH, Selb, Germany), and the temperature range was set between 0 °C and 80 °C with a heating/cooling rate of 10 °C min^−1^. Thermal stability was assessed using a TAG Q500 instrument (TA Instruments—Waters LLC, New Castle, DE, USA), by measuring the weight change in the sample within the temperature range of 30 °C to 600 °C at a heating rate of 10 °C min^−1^ under a nitrogen atmosphere.

### 2.5. Performance Measurements

#### 2.5.1. Loading Amount and Leakage Test

The PCM loading amount (ω) in carbon-based support was calculated according to Equation (1) [31]:

(1) $$\omega =\frac{m_{1}-m_{2}}{m_{1}}\times 100\%$$

where *m*_1_ is the total mass of SSPCMs, g; *m*_2_ is the mass of the biochar skeleton, g.

Leakage tests were conducted on the samples that had been placed on filter papers [32]. After holding at 60 °C for 2 h, the samples could be monitored for leakage. If the liquid traces were detected on filter papers, the sample had a leak problem.

#### 2.5.2. The Thermal Response Simulation Experiment

**Figure 3:**
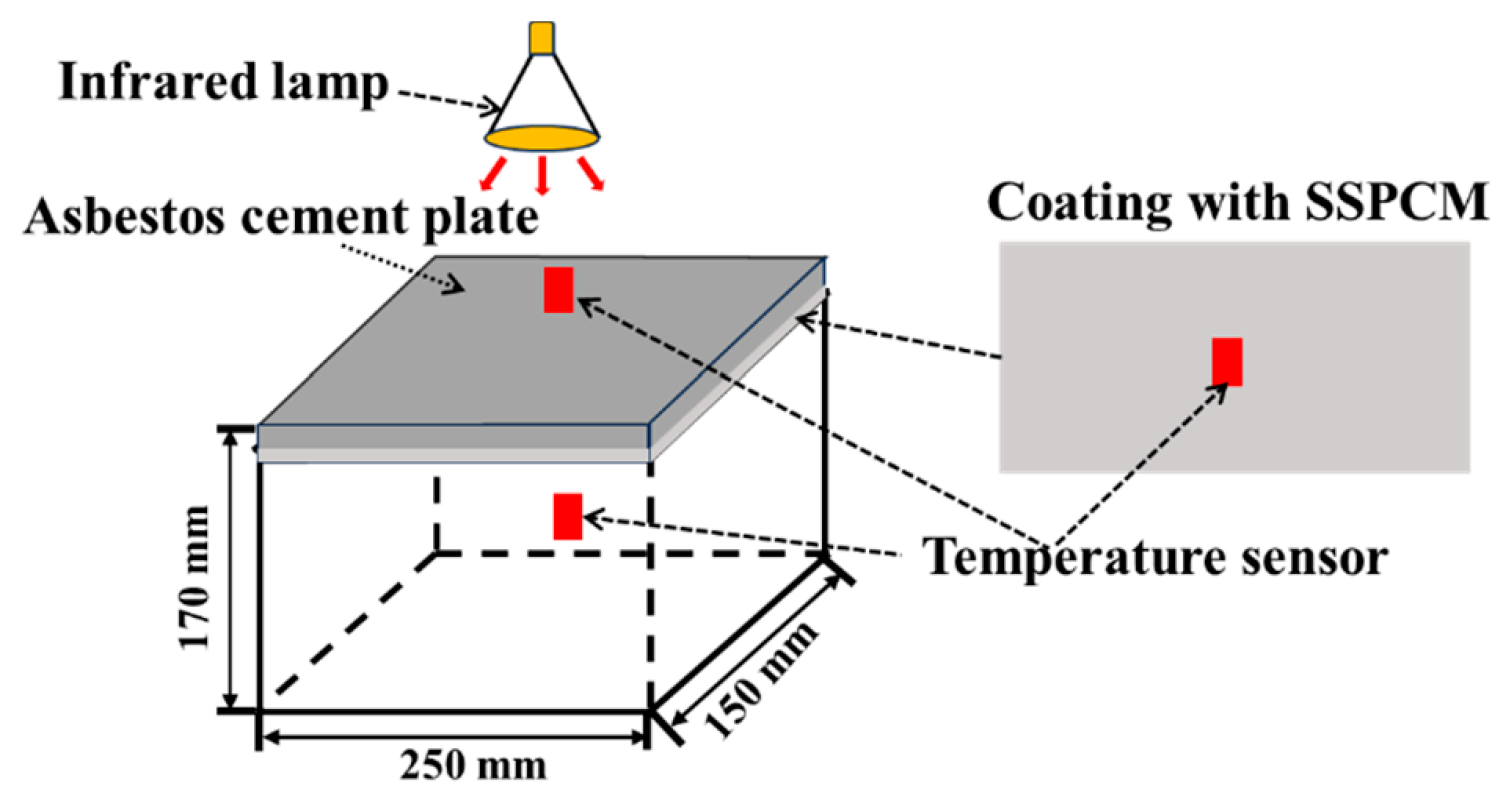
The thermal response simulation experimental device.

Thermal energy storage application in buildings was evaluated using a thermal response simulation experimental device (Figure 3). First, composite phase change coatings with SSPCM contents of 5, 10, 15, 20, and 25 wt% were prepared. Namely, KCB/LA-MA was blended with putty powder and distilled water to formulate a uniform and viscous mixture. To ensure uniformity, the dry components were first stirred thoroughly, followed by the dropwise addition of distilled water under continuous stirring until a smooth, thixotropic paste was obtained. The same total mass of this mixture was applied to every pre-cleaned asbestos cement board with a spatula, and the surface was leveled by repeated scraping. For comparison, a plain putty coating without SSPCM was applied to reference boards. All coated cement boards were dried in an oven at 30 °C for 2 h.

Subsequently, the top surface of the self-fabricated test setup was formed by the coated cement board, while the surrounding walls were insulated with extruded polystyrene panels for thermal performance testing. The estimated areal SSPCM loadings on the upper surface were 83, 167, 250, 333, and 417 g·m^−2^ for the 5, 10, 15, 20, and 25 wt% coatings, respectively. Finally, a 150 W infrared lamp was used to heat the asbestos cement plate coated with SSPCM. The model chamber was irradiated with an infrared lamp until the indoor temperature reached a certain value, after which the irradiation was stopped. The temperature was simultaneously monitored using a Pt100 platinum resistance sensor with an accuracy of ±0.1 °C.

## 3. Results and Discussion

### 3.1. Pore Structure and Microscopic Morphology of Biochar and SSPCMs

#### 3.1.1. Pore Structure Analysis

**Figure 4:**
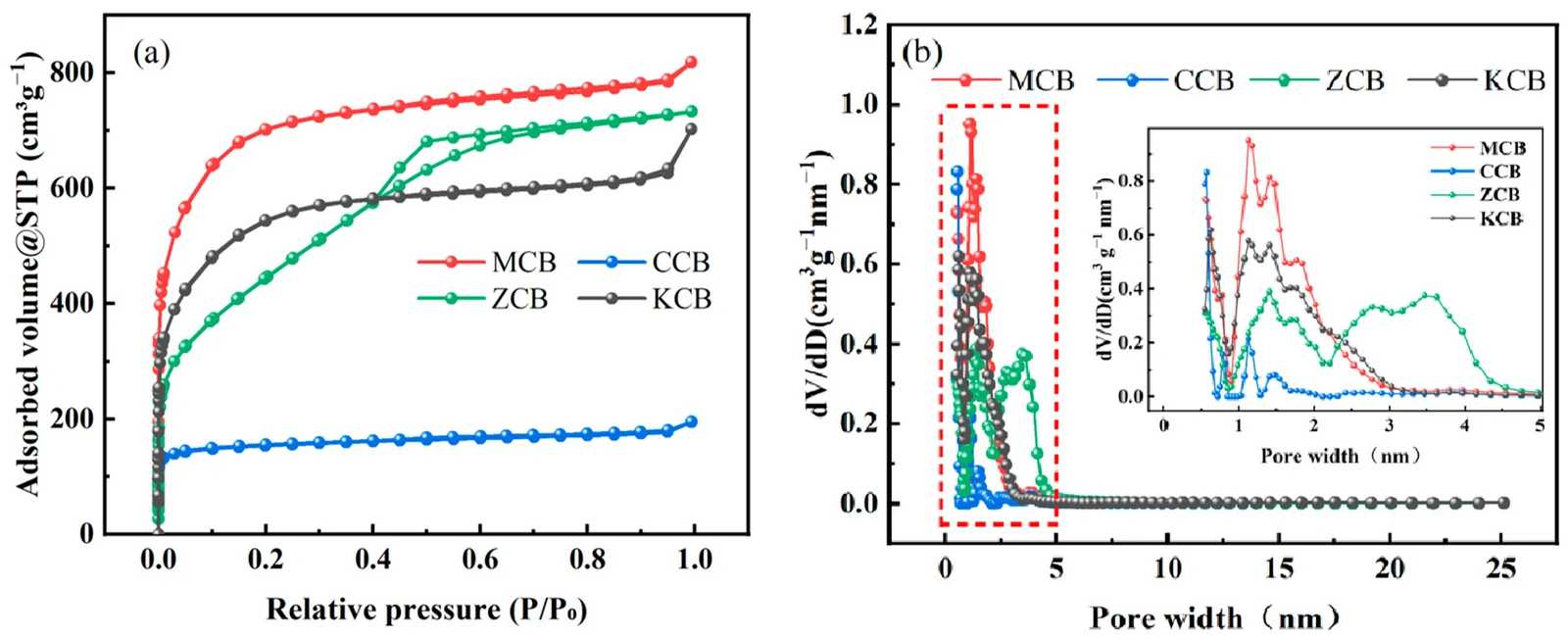
(**a**) N_2_ adsorption–desorption isotherms; (**b**) pore size distribution curves of the prepared samples (CB-to-agent mass ratio of 1:3). MCB, CCB, ZCB, and KCB denote the samples prepared with MgCl_2_, CaCO_3_, ZnCl_2_, and KOH, respectively.

The pore structure of the skeleton material was characterized by N_2_ adsorption–desorption isotherms (shown in Figure 4) in order to analyze the reasons for the efficient encapsulation of PCM. According to the IUPAC classification, KCB, MCB and CCB exhibit a typical I-type adsorption isotherm. The isotherms of KCB and MCB show drastic N_2_ adsorption phenomenon at low relative pressure (P/P_0_ < 0.01), indicating abundant micropores. A gradual increase in N_2_ uptake and a hysteresis loop are observed for KCB at high relative pressure conditions (P/P_0_ > 0.4), which proves the presence of mesoporous structure. Combined with the pore size distribution analysis, KCB presents a highly advantageous pore structure characterized by ultramicropores/micropores (<2 nm) and narrow mesopores (2–3 nm), which may be particularly favorable for PCM encapsulation. By contrast, the N_2_ adsorption–desorption isotherm of ZCB shows a typical Type IV with a pronounced hysteresis loop over the P/P_0_ range of 0.4–0.9, which is characteristic of capillary condensation in mesopores. This indicates that ZnCl_2_ activation effectively promotes mesopore development, yielding a pore size distribution mainly within 2–10 nm.

Pore structure parameters of activated biochar are summarized in Table 3. MCB possesses an exceptionally high specific surface area with 2561 m^2^ g^−1^, but its relatively small average pore diameter (2.0 nm) may induce significant steric hindrance to PCM impregnation. ZCB exhibits the largest total pore volume (1.32 cm^3^ g^−1^) and average pore diameter (2.7 nm), but excessively large pores are unfavorable for immobilizing molten PCM through capillary forces. Compared with these samples, KCB combines the characteristics of a high specific surface area (1946 m^2^ g^−1^) and an average pore diameter of 2.2 nm. This average pore size is slightly larger than the molecular size of LA (~1.83 nm) and MA (~1.91 nm) [33], indicating favorable pore-molecule size matching for effective encapsulation. Relative to the broader mesopores of ZCB, the narrower confined spaces in KCB can more effectively “lock” PCM molecules into the carbon skeleton, thus suppressing molecular migration and leakage during the solid–liquid phase transitions. Accordingly, KCB is identified as the optimal supporting matrix for encapsulating organic PCMs.

#### 3.1.2. Morphological Characteristics

**Figure 5:**
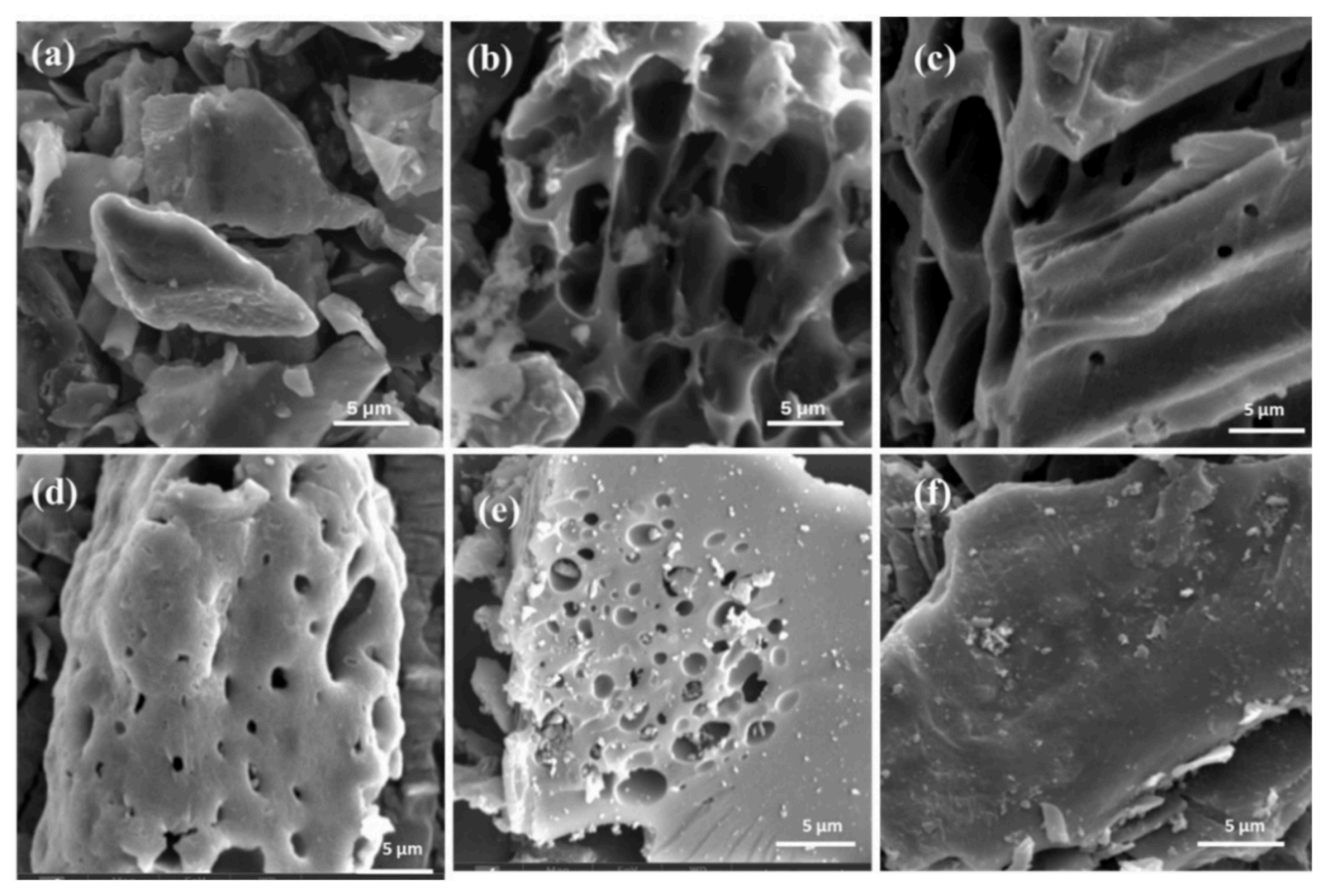
SEM images of (**a**) CB, (**b**) KCB, (**c**) MCB, (**d**) CCB, (**e**) ZCB, and (**f**) KCB/LA-MA, prepared with a CB-to-agent mass ratio of 1:3.

SEM analysis further reveals distinct surface morphologies for biochar activated with different agents. The micrographs of several skeleton materials and KCB/LA-MA are shown in Figure 5. As observed in Figure 5a, CB exhibits a compact morphology with almost no discernible pores, featuring only irregular fissures and concave surfaces. After activation, pore structures appear on the surfaces of all the activated carbons. KCB displays a well-developed and honeycomb-like pore structure, which is beneficial for PCM loading. MCB has a highly developed surface with deep wrinkle-like ravines; whereas, CCB appears relatively compact and smooth, with only a few shallow and isolated pit-like pores, suggesting a relatively weak activation effect of CaCO_3_. ZCB displays a surface with circular pores of various sizes, forming a bubble-burst-like morphology, which can be attributed to the strong dehydration and pore-forming capability of ZnCl_2_ during high-temperature activation [34]. As shown in Figure 5f, the surface of the KCB/LA-MA composite appears relatively smooth, which is attributed to vacuum impregnation of the PCM into KCB, indicating that the eutectic PCM has been effectively loaded within the porous carbon support.

### 3.2. Encapsulation Effects of SSPCMS and Form-Stable Performance

**Figure 6:**
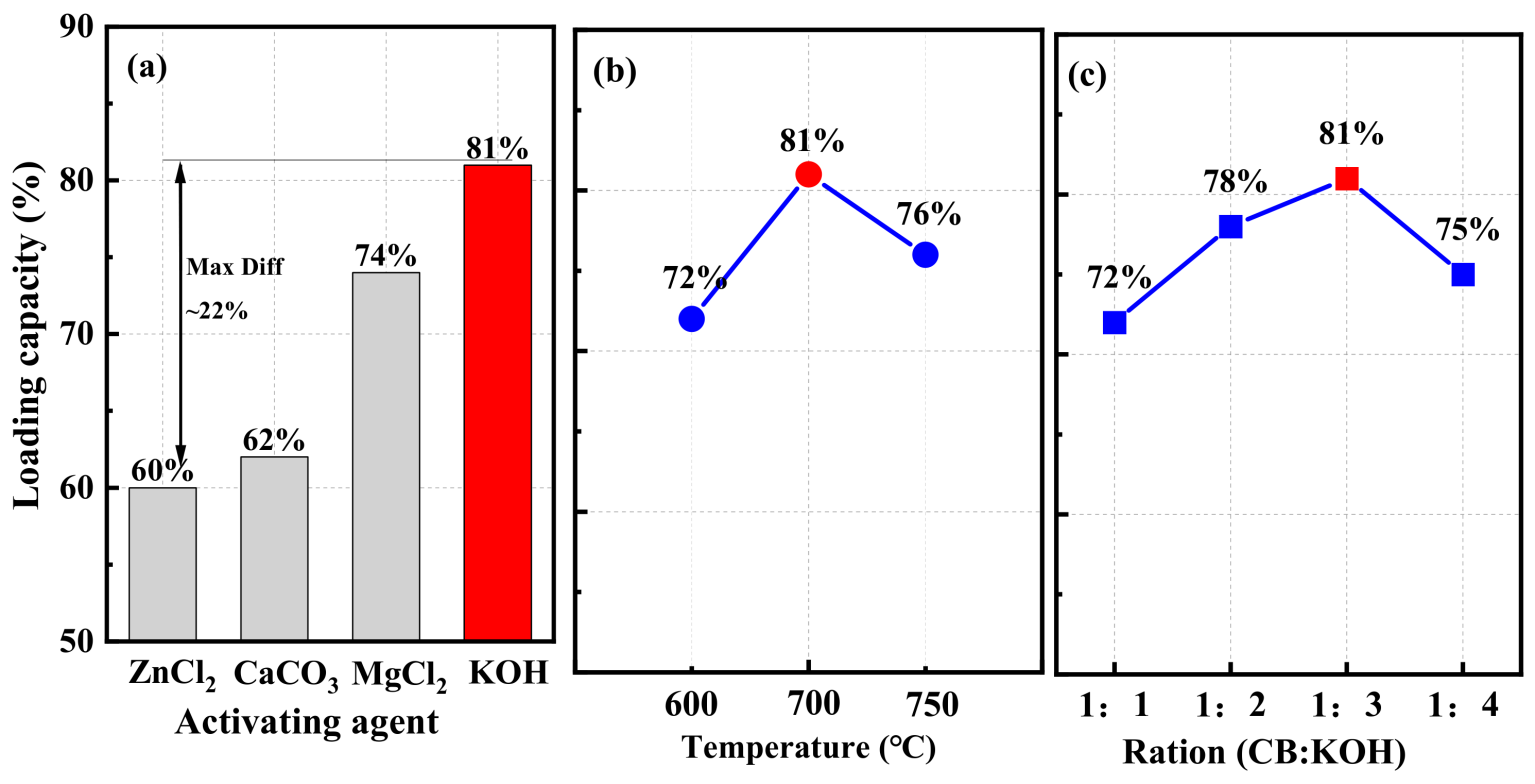
Effects of preparation conditions on the loading capacity of coconut shell activated carbon: (**a**) CB-to-agent mass ratio of 1:3 at 700 °C; (**b**) temperature variation at a CB: KOH mass ratio of 1:3; (**c**) CB: KOH mass ratio variation at 700 °C.

Effective encapsulation in composite SSPCMs is crucial to achieve a high loading amount while preventing leakage of the molten PCM. The PCM loading capacities of biochar under different activation conditions are shown in Figure 6. Biochar (CB) without activation has a loading capacity of 59%, while KOH-activated biochar (KCB) achieves the highest PCM loading capacity of 81%, which is nearly 22% higher than that of ZnCl_2_-activated biochar (ZCB, 60%). This comparison suggests that the choice of activating agent significantly affects the loading performance. Using KOH as an activator, comparative analysis also indicates that variations in activation temperature and dosage produce only marginal changes in PCM loading capacity, with the overall variation remaining within approximately 9%. This result suggests that the chemical nature of activating agents plays a critical role in regulating the pore structure and surface functional groups of the porous carbon, which influence its ability to accommodate and retain PCM. In addition, the markedly improved loading capacity of KCB indicates that KOH activation may generate a more suitable porous framework or surface characteristic for effective PCM encapsulation, thereby enhancing the storage capacity of the SSPCM.

**Figure 7:**
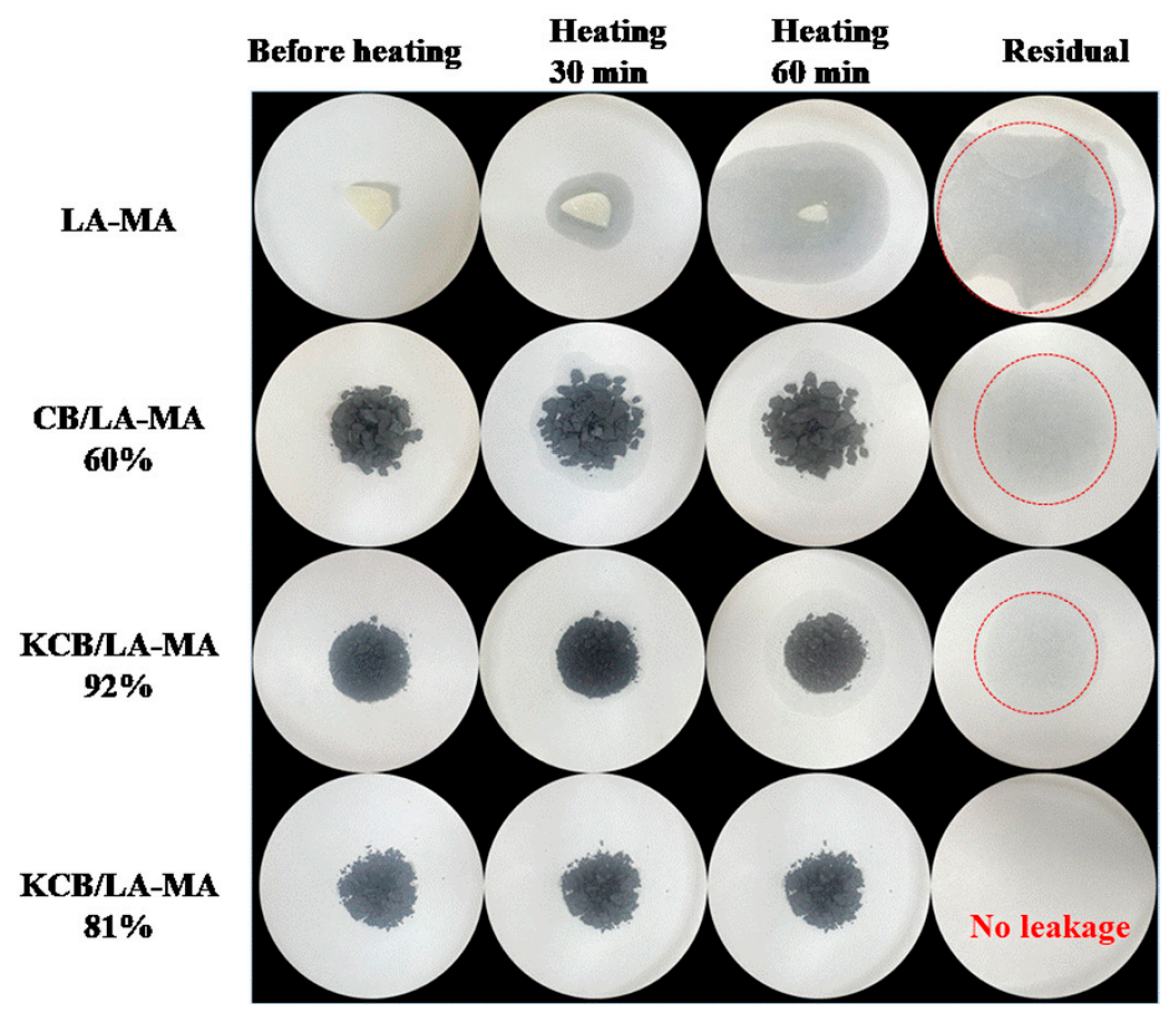
Comparison of leakage for LA-MA, CB/LA-MA, and KCB/LA-MA (KCB, a CB: KOH mass ratio of 1:3) before and after heating at 60 °C.

A key problem faced by organic PCMs in practical applications is the possible leakage during the phase change process, which greatly limits their popularity and application scope. In order to evaluate the practical application performance of carbon-based SSPCMs, leakage has become an important evaluation indicator, and the results of composite phase change materials are shown in Figure 7. After heating for 30 min, LA-MA had melted, and slight liquid stains attributable to leakage were observed on the filter paper beneath the CB/LA-MA (loading 60% PCM) and the KCB/LA-MA with 92% PCM mass content. After heating for 60 min, LA-MA was almost completely molten, and the stained areas for CB/LA-MA and KCB/LA-MA (92%) became more pronounced. In contrast, the KCB/LA-MA with an 81% loading exhibited no observable leakage even after 60 min of heating, maintaining excellent shape stability. These results indicate that the porous structure and functional groups of the carbon skeleton could provide sufficient space and binding sites to adsorb and retain the molten PCM throughout the phase-transition process.

### 3.3. Surface Characteristics

#### 3.3.1. XPS Analysis

**Figure 8:**
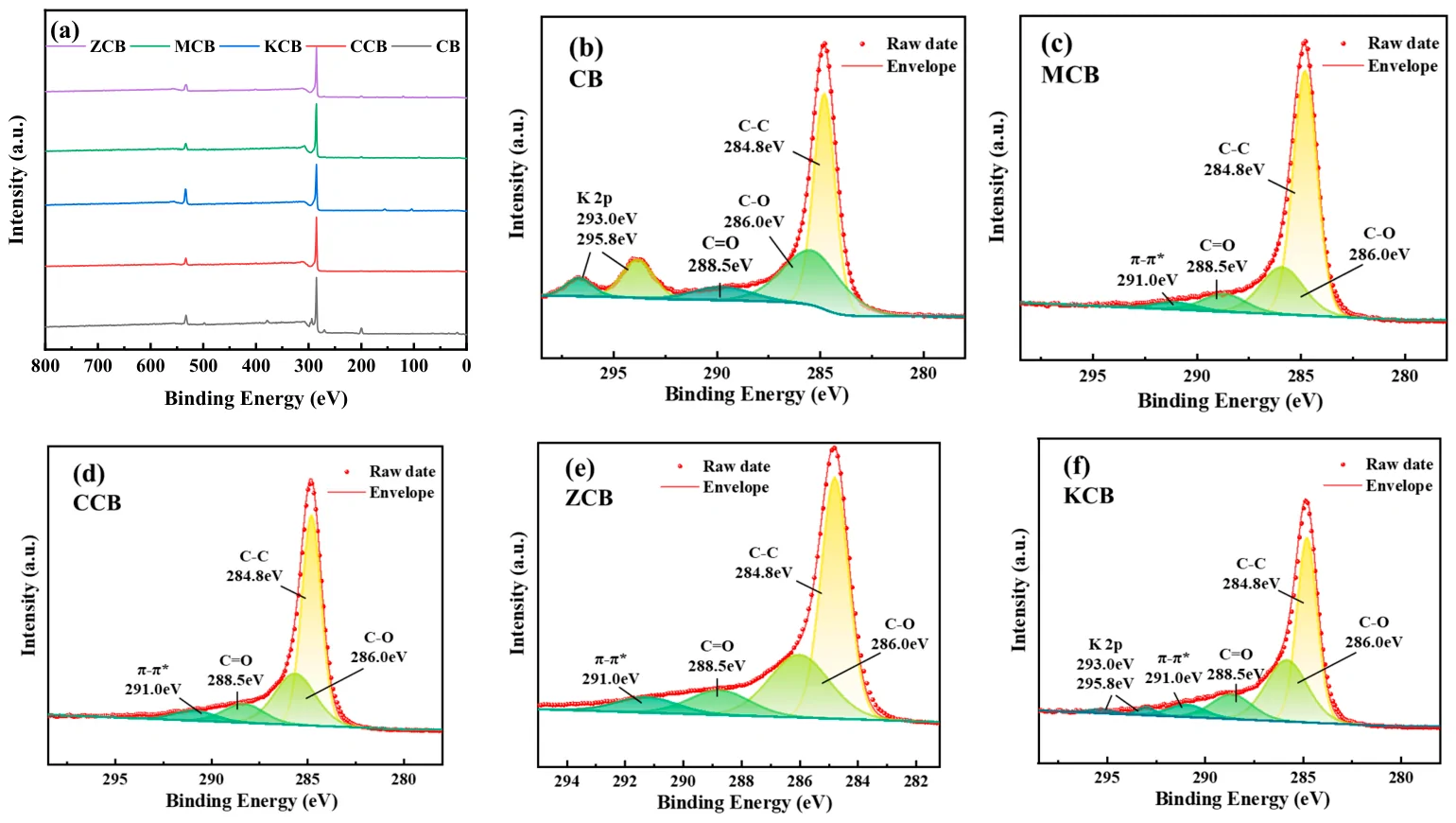
(**a**) XPS survey spectra; (**b**–**f**) C1s high-resolution spectra of the activated carbons at a CB-to-agent mass ratio of 1:3.

To elucidate the surface characteristics of the activated carbon supports, XPS analysis was performed. The results are presented in Figure 8, and Table 4 lists the proportion of groups in the C1s spectra of different carbons. The spectra in Figure 8a show that all samples are dominated by C and O elements, but their relative contents varied significantly. Among them, KCB exhibits the strongest O 1s signal, indicating the highest abundance of oxygen-containing functional groups on its surface.

As shown in the C 1s spectrum of CB, the conventional characteristic peaks at 286.0 eV and 288.5 eV are assigned to C-O and C=O, respectively. In addition, two peaks located at 293.0 and 295.8 eV are observed, which can be attributed to the K 2p orbital signals of residual mineral species derived from the biomass precursor [35]. Notably, both MCB and CCB exhibit a broad weak peak near 291.0 eV, corresponding to the π-π* shake-up satellite peak associated with aromatic carbon structures [36]. Compared with CB, the C-O and C=O signals remained at moderate intensities, reflecting the relatively mild surface modification induced by these activating agents. A distinct π-π* satellite peak is also observed at approximately 291.0 eV in the C 1s spectrum of ZCB, suggesting the presence of a conjugated aromatic structure. Additionally, the sum of relative proportions of C-O and C=O components is relatively high (~30%).

KCB presents the highest proportions of the sum of C-O and C=O species (~33%), which can serve as key anchoring sites for PCM. In addition, a very weak peak at 293.0 eV is assigned to the K 2p signal, while the broad feature near 291.0 eV is attributed to the π–π* satellite peak. The high density of O-containing functional groups on KCB surface provides abundant active sites, which are highly favorable for improving the interfacial wettability between the molten PCM and the carbon matrix, while simultaneously promoting the formation of a stable hydrogen-bonding network with PCM molecules. This oxygen-rich surface performance, together with the favorable pore structure, makes KCB an ideal support for the fabrication of high-performance SSPCM.

#### 3.3.2. FTIR Analysis

**Figure 9:**
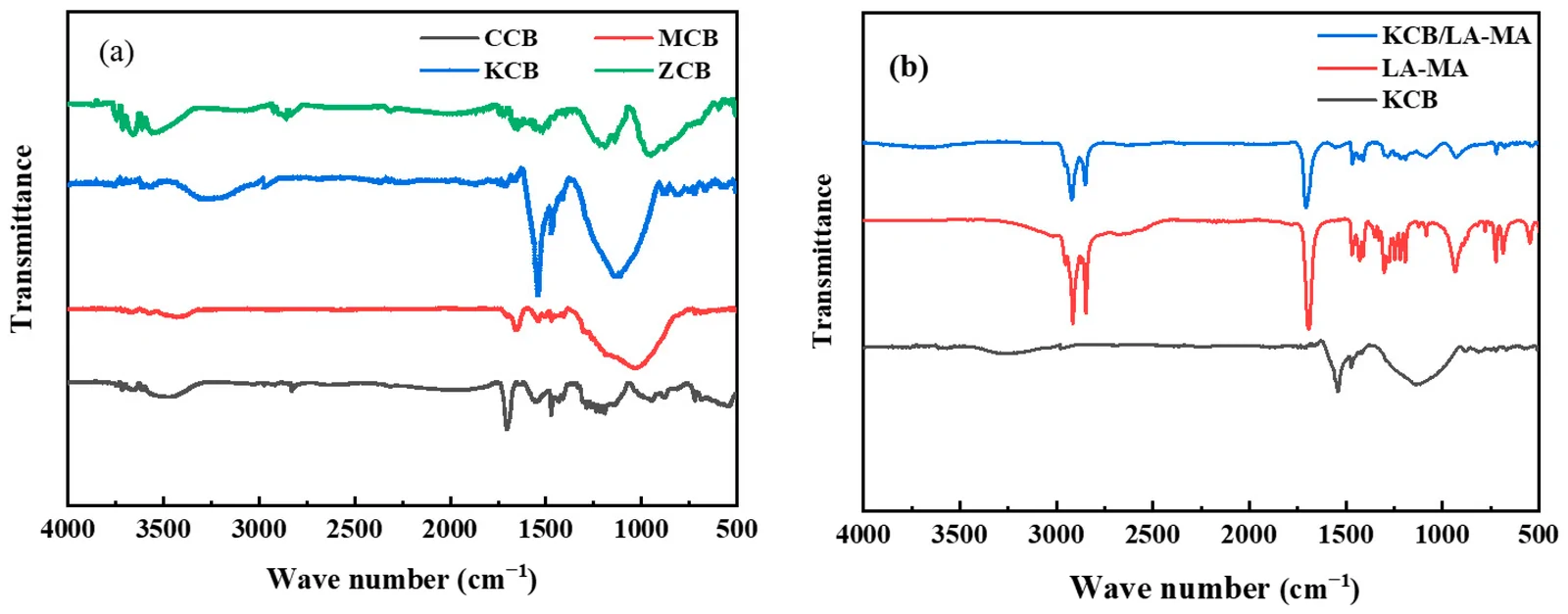
FTIR spectra of the activated carbons at a CB-to-agent mass ratio of 1:3. (**a**) Activated carbon; (**b**) SSPCMs with LA-MA.

To further clarify the effect of different activating agents on the surface characteristics of biochar, FTIR analysis was discussed, and the results are shown in Figure 9. Comparative analysis demonstrates that KCB shows an intense characteristic C-O absorption band in the range of 1000~1300 cm^−1^ and C=C/C=O stretching vibration at 1601 cm^−1^, indicating that KOH activation may introduce a high density of oxygen-containing active sites onto the carbon framework. Chemical compatibility between the skeleton and the PCM is crucial for extending the service life of SSPCMs. FTIR spectroscopy is usually used to evaluate the chemical compatibility of constituent materials [37].

The FTIR spectra of LA-MA and KCB/LA-MA are presented in Figure 9b. The spectrum of LA-MA exhibits the bands at 2918 cm^−1^ and 2850 cm^−1^ (C–H stretching vibration [38]), 1700 cm^−1^ (C=O stretching vibration [39]), 1468 cm^−1^ (–CH_2_ bending vibration), 1302 cm^−1^ (in-plane bending vibration of –OH), 939 cm^−1^ (out-of-plane deformation vibration of –OH), and 723 cm^−1^ (out-of-plane bending vibration of C–H). Notably, for KCB/LA-MA, the spectrum still shows distinct peaks, such as C=O stretching band at ~1700 cm^−1^ and C-O stretching bands in the range of 1000~1200 cm^−1^ [40]. These results indicate the successful incorporation of LA-MA into skeleton material. Moreover, compared with the FTIR spectrum of the pure phase change material, no new absorption peaks appeared in the spectrum of the SSPCM. This result indicates that the skeleton material and LA-MA are physically mixed and present excellent chemical compatibility.

### 3.4. Thermophysical Properties and Thermal Reliability

**Figure 10:**
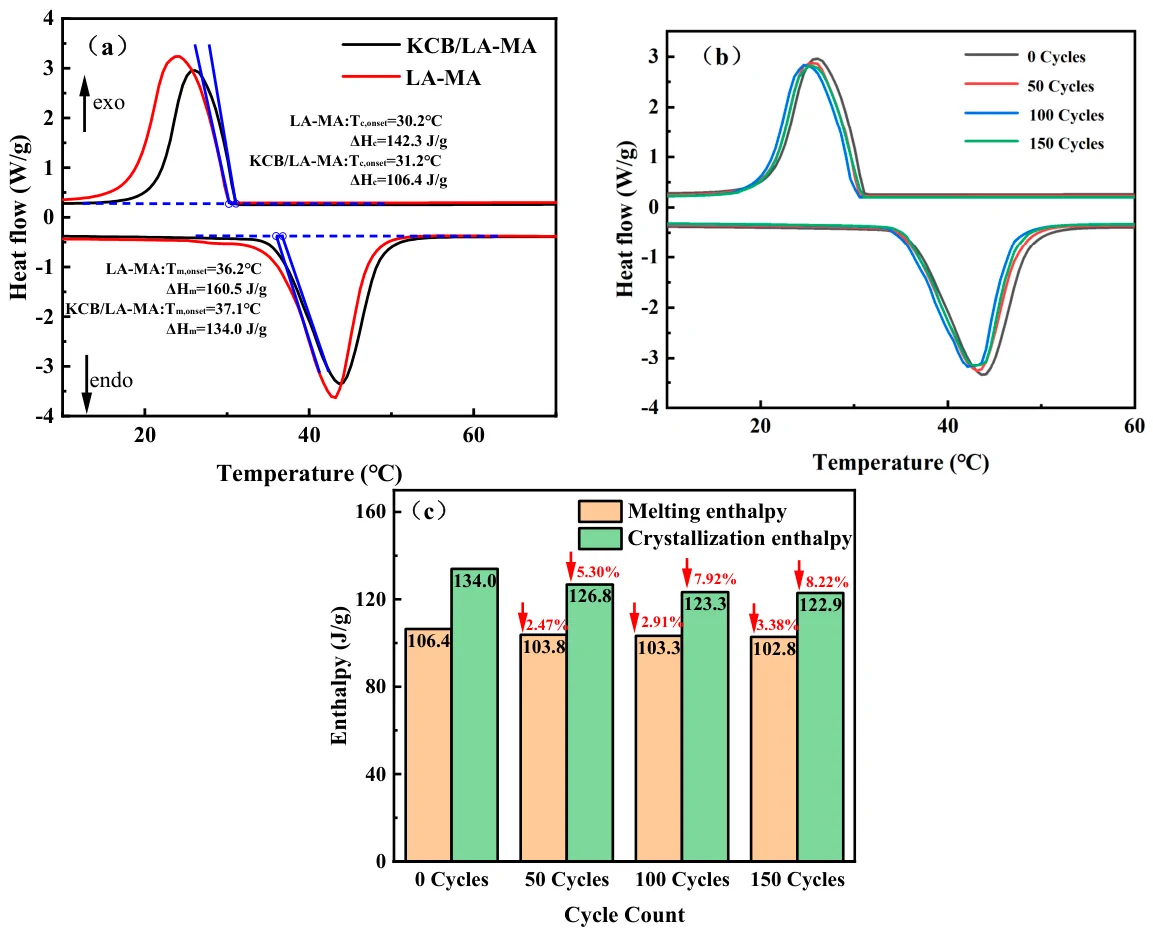
(**a**) DSC curves of KCB/LA-MA and LA-MA; (**b**) DSC curves of KCB/LA-MA after different thermal cycle numbers; (**c**) variations in melting/crystallization enthalpies.

Phase transition temperature and enthalpy are important factors affecting the performance of PCMs, and the DSC curves are presented in Figure 10. The DSC thermograms indicate that LA-MA exhibits a set of single endothermic/exothermic peaks with the melting/crystallizing points of 36.2 °C/30.2 °C, and the melting and freezing enthalpies are 160.5 J g^−1^ and 142.3 J g^−1^, respectively. For SSPCM, the melting/crystallizing points slightly shift to 37.1 °C/31.2 °C. The slight shift in temperature observed for the SSPCM could be attributed to the interaction between surface functional groups on the supporting matrix and the PCM, which can increase the intrinsic energy barrier during melting and thereby elevate the melting temperature of the composite PCM [24].

The melting enthalpy of KCB/LA-MA is 134.0 J g^−1^ and the freezing enthalpy is 106.4 J g^−1^. Compared with those of the pristine PCM, the enthalpy values of the SSPCM are reduced, primarily because incorporation of biochar decreases the mass fraction of the PCM in the composite, while the biochar itself does not undergo any phase transition within the operating temperature range. Table 5 compares the thermal properties between the prepared SSPCM and other shape-stabilized PCMs reported in the literature. Nevertheless, despite the decrease in enthalpy, KCB/LA-MA still exhibits a comparatively high latent heat for thermal energy storage.

Thermal cycling stability is one of the most important indicators for evaluating the long-term reliability of SSPCMs in practical building energy-saving applications. KCB/LA-MA was subjected to 150 heating/cooling cycles to assess durability, and the DSC curves after 0, 50, 100 and 150 thermal cycles are presented in Figure 10b. With increasing cycle number, the melting and crystallization peaks remain essentially unchanged, and the peak positions show negligible shift, indicating excellent thermal reversibility. After 150 cycles, the melting enthalpy decreases from 106.4 to 102.8 J g^−1^, corresponding to a loss of only 3.38%, while the crystallization enthalpy decreases from 134.0 to 122.9 J g^−1^, representing a loss of 8.22%. These results indicate that KCB/LA-MA with excellent thermal cycling reliability has the potential for reliable operation in building envelope thermal management.

**Figure 11:**
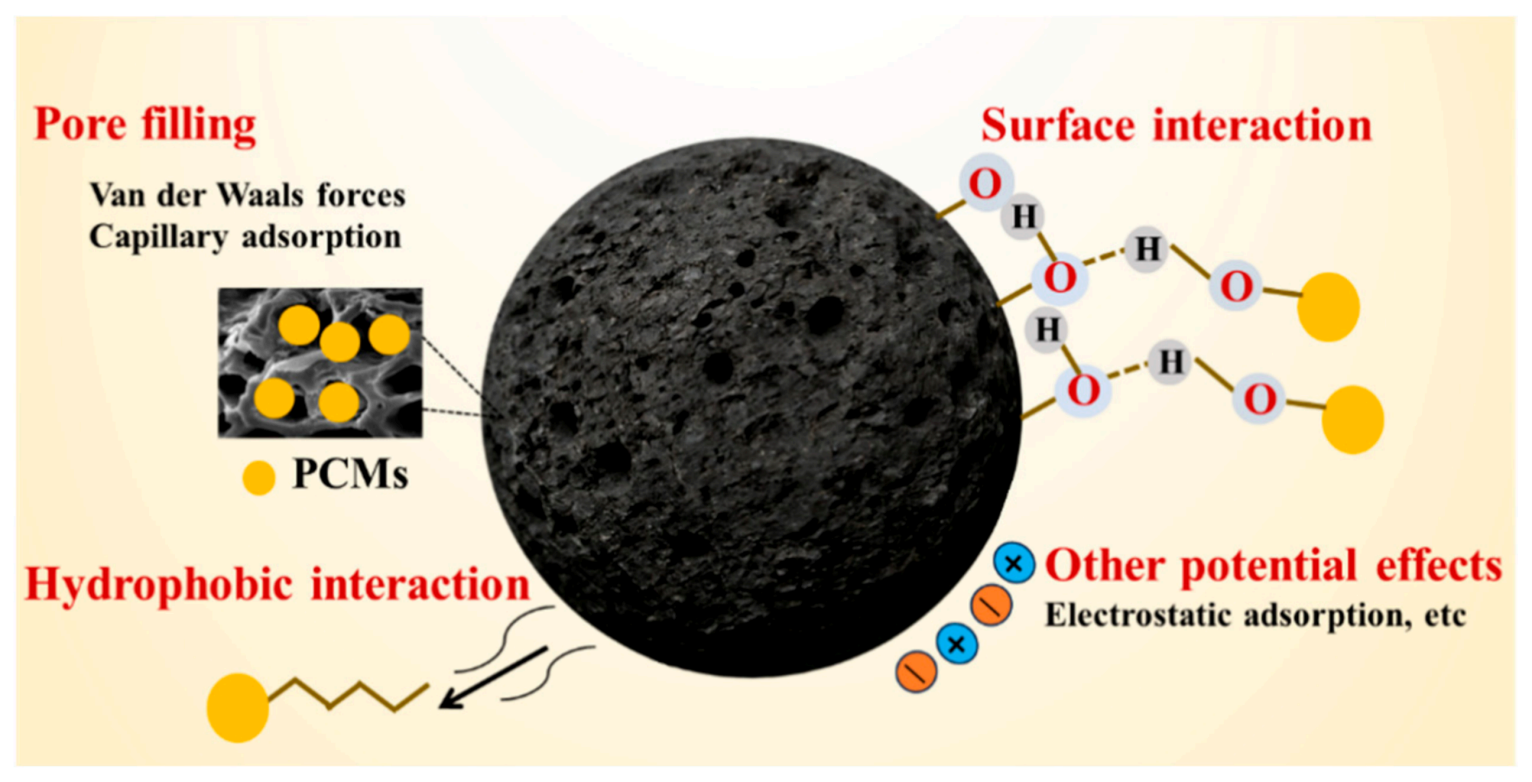
Schematic of the adsorption and stabilization mechanisms of LA-MA on the activated biochar support.

KCB/LA-MA composite phase change material possesses excellent shape stability and thermal properties, which are related to the characteristics of the biochar matrix, as confirmed by the pore structure analysis, XPS and FTIR analyses. As shown in Figure 11, the activated biochar, especially KCB, provides a well-developed pore structure composed of abundant micropores and suitably sized mesopores, which offers a large accessible surface area and pore channels for the molten fatty-acid molecules. Under capillary driving forces, molten LA-MA penetrates into the internal pore channels of the carbon matrix, where it is subsequently retained by strong capillary forces generated within the confined pore space [47]. This confinement is essential for preventing the leakage of the PCM during the melting–solidification process.

In addition to this pore-filling physical adsorption, surface interactions may further contribute to the loading amount and stability of LA-MA. In particular, the oxygen-containing functional groups on the biochar surface can form hydrogen-bonding interactions with the carboxyl groups of LA-MA. These specific interactions are stronger than van der Waals attractions and may thus enhance not only PCM loading capacity but also the thermal stability of the composite. Besides these, fatty acids possess long nonpolar alkyl chains, while the carbon skeleton of biochar is relatively hydrophobic, which may promote molecular spreading on the carbon surface, thereby improving impregnation capacity. Other weak interactions, such as those between aromatic domains of biochar and the hydrocarbon chains of fatty acids, may also contribute to stabilization [48]. Overall, these biochar–PCM interactions, in combination with the pore-filling physical adsorption discussed above, not only enhance the loading capacity of LA-MA but may also influence the melting and solidification processes of the PCM within the biochar, thereby affecting its thermal performance.

### 3.5. Thermal Stability

**Figure 12:**
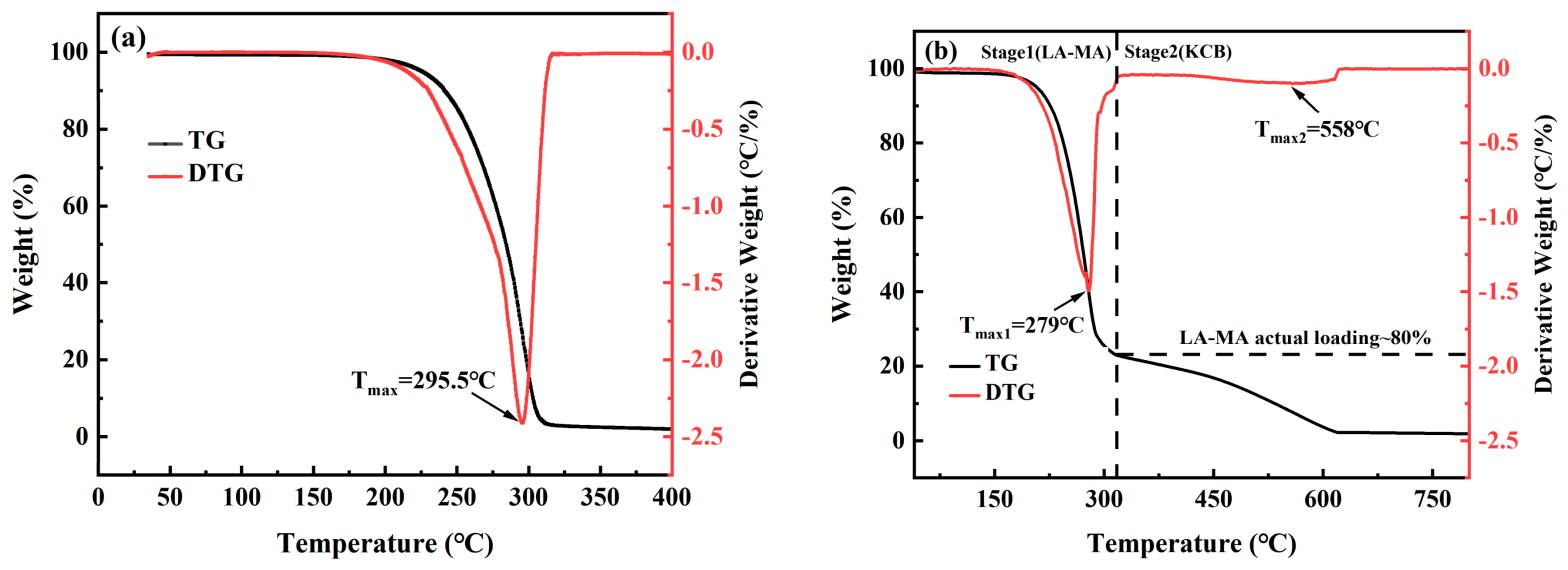
TGA curves and DTG curves. (**a**) LA-MA; (**b**) KCB/LA-MA.

Thermal stability is also critical for the practical application of composite materials. TG analysis was conducted to evaluate the high-temperature thermal stability of the eutectic PCM (LA-MA) and KCB/LA-MA under a nitrogen atmosphere, as shown in Figure 12. The main mass loss stage of LA-MA occurs between 150 °C and 320 °C, which is attributed to the decomposition of fatty acid. TG and DTG curves of KCB/LA-MA can be divided into two main stages. The first stage corresponds to the degradation of the LA-MA component, with the maximum mass loss rate occurring at 279 °C. The second stage is associated with the thermal decomposition of the KCB matrix. These results indicate that the onset decomposition temperatures of both LA-MA and KCB/LA-MA are higher than 100 °C. Such temperatures are unlikely to be encountered under typical building service conditions, demonstrating that the material exhibits good thermal stability and is therefore suitable for use in building envelope applications.

### 3.6. Thermal Energy Storage Application

**Figure 13:**
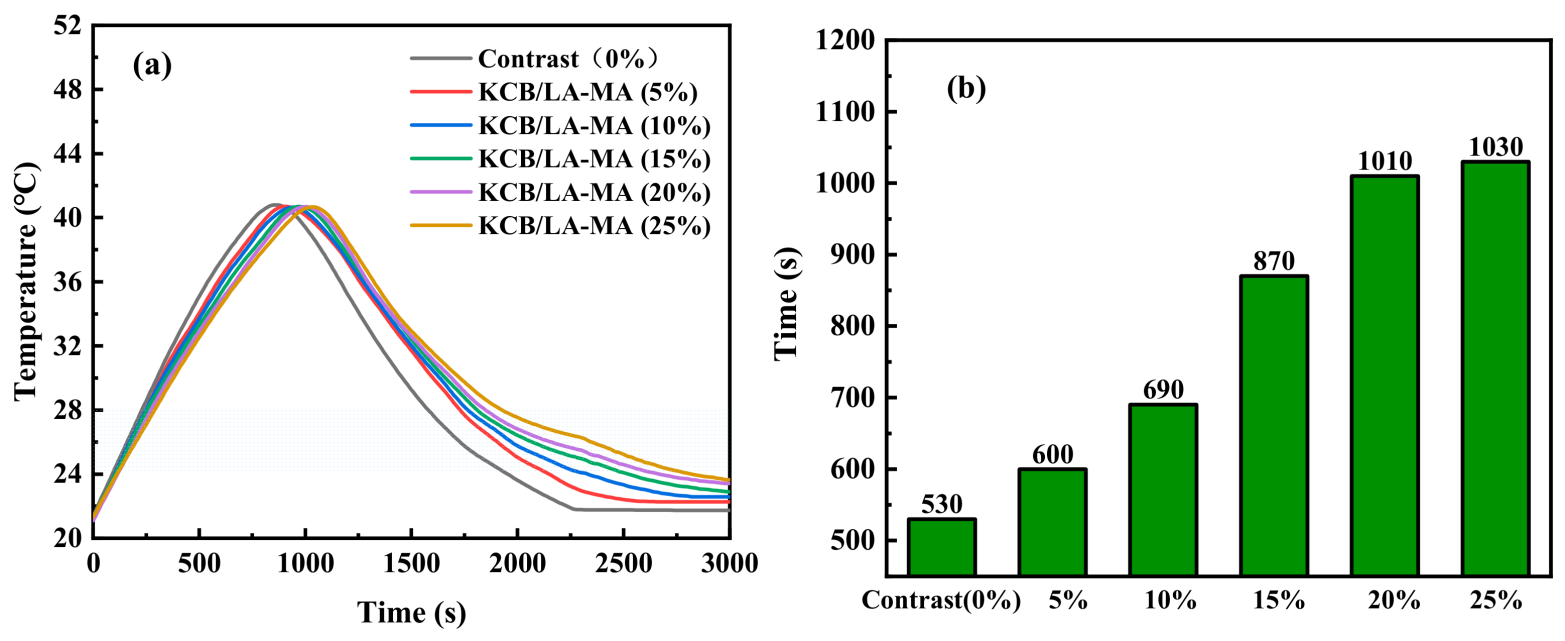
(**a**) Indoor temperature change curves of a model chamber coated with KCB/LA-MA composites at different mass fractions; (**b**) duration of the thermal response during which the indoor temperature remained within the range of 24~28 °C.

Thermal energy storage for buildings is a potential application of PCMs [49]. To enable application in buildings, the mixture of as-prepared KCB/LA-MA and plain putty was coated with a cement board, in the comparison with a board coated with plain putty as the control group. The model chamber was irradiated with an infrared lamp until the indoor temperature reached 40 °C, then the irradiation was stopped. As displayed in Figure 13a, the time required for each group to reach 40 °C increases with the addition of KCB/LA-MA. During the heating stage, the indoor temperature–rise curve of the control group exhibits the steepest slope under the light irradiation, reaching 40 °C at 850 s. Conversely, the temperature–rise curves of the KCB/LA-MA groups increase relatively slowly. The model chamber covered with the coating containing 25% KCB/LA-MA requires approximately 1050 s to reach the target temperature (40 °C). During infrared irradiation, KCB/LA-MA undergoes a phase change and absorbs heat, thereby leading to a relatively slower increase in indoor temperature. After removing the light irradiation, the temperature of the KCB/LA-MA groups dropped slowly compared with the control group, making the cooling process more stable and reducing the fluctuation range of inside temperature. This is mainly attributable to the release of a large amount of latent heat during the cooling process. Figure 13b presents the duration during which the indoor temperature remained within the range of 24~28 °C, in accordance with human thermal comfort requirements. Compared with the control group, the time is significantly prolonged at a KCB/LA-MA content of 25 wt%. Thus, carbon-based SSPCM with LA-MA can absorb light energy and gradually release heat during thermal cycles, thereby helping to maintain a relatively stable indoor temperature and contributing to building energy conservation.

## 4. Conclusions

Carbon-based porous matrices derived from waste biomass serve as effective structural frameworks for PCM, addressing leakage and low loading capacity. Compared with ZnCl_2_- and MgCl_2_-activated biochars, the KOH-activated biochar (KCB) offers a high specific surface area (1946 m^2^ g^−1^) and a microporous/narrow-mesoporous structure matching PCM molecule sizes, enabling the highest loading capacity (81%) for the LA-MA binary eutectic. The resulting KCB/LA-MA composite exhibits good shape stability, high phase change latent heat, and thermal stability up to 100 °C. After 150 thermal cycles, it shows only a 3.38% reduction in heat storage capacity. Its excellent performance is attributed to pore filling and the interactions (e.g., hydrogen bonding) between the fatty acids and the oxygen-containing functional groups on the biochar surface. In a simulated application, a KCB/LA-MA-containing coating slows the rates of heating and cooling, helping maintain a relatively constant indoor temperature. This work provides a sustainable approach for fabricating high-performance composite SSPCMs from biomass-derived porous carbon, although the potential for further optimization of individual activating agents is acknowledged and left for future work.

## Figures and Tables

**Table 1:** Properties of phase change materials.

Sample	Melting Point (°C)	Enthalpy of Fusion (J g^−1^)	Molar Enthalpy of Fusion (kJ mol^−1^)
LA	43.9	186.2	37.3
MA	53.8	200.1	45.7

**Table 2:** Proximate analysis of raw coconut shell biomass.

Sample	Proximate Analysis (wt%, Air-Dry Basis)			
	Moisture	Ash	Volatile Matter	Fixed Carbon
Coconut shell	9.71	2.69	70.45	17.15

**Table 3:** Pore structure parameters of biochar samples treated with different activating agents at a CB-to-agent mass ratio of 1:3. MCB, CCB, ZCB, and KCB correspond to the biochars prepared with MgCl_2_, CaCO_3_, ZnCl_2_, and KOH, respectively.

Samples	S_BET_ (m^2^ g^−1^)	V_total_ (cm^3^ g^−1^)	D_average_ (nm)
MCB	2561	1.27	2.0
CCB	597	0.30	2.0
ZCB	1952	1.32	2.7
KCB	1946	1.09	2.2

**Table 4:** The proportion of groups in C 1s spectra of different activated carbons at a CB-to-agent mass ratio of 1:3.

Group Type	Binding Energy (ev)	Proportion (%)				
		CB	MCB	CCB	ZCB	KCB
C-C	284.8	73.66	68.38	69.93	66.28	63.14
C-O	286.0	19.15	17.78	14.77	23.20	20.99
C=O	288.5	7.18	10.00	11.36	7.54	12.31
π-π*	291.0	-	3.85	3.93	2.98	3.55

**Table 5:** The comparison of thermal properties of SSPCMs with fatty acid as PCM in the literature.

Biomass	PCM	Loading Amount (%)	Peak Melting Temperature (°C)	Melting Enthalpy (J g^−1^)	References
Chili straw	Palmitic acid	50	65.7	95.5	[41]
Pinecone	Palmitic acid	60	59.25	84.74	[42]
Sunflower	Palmitic acid	-	67.65	207.9	[43]
Corn straw	Stearic acid	65.5	50.99	114.17	[44]
Wheat bran	Stearic acid	-	70.7	56.96	[45]
Melon-seed shells	Stearic acid	75	58.34	149.57	[46]
Waste rice	Palmitic acid and lauric acid	78.8	34.3	135.4	[23]
Coconut shell	Lauric acid and myristic acid	81	37.1	134.0	This work

## Data Availability

The original contributions presented in this study are included in the article. Further inquiries can be directed to the corresponding author.

## Abbreviations

The following abbreviations are used in this manuscript:

PCMs	Phase change materials
SSPCMs	Shape-stabilized phase change materials
LA-MA	Lauric acid–myristic acid binary eutectic
BET	Brunauer–Emmett–Teller
DFT	Density functional theory
IUPAC	International Union of Pure and Applied Chemistry
